# Oxidized Low-Density Lipoprotein Accumulation in Macrophages Impairs Lipopolysaccharide-Induced Activation of AKT2, ATP Citrate Lyase, Acetyl–Coenzyme A Production, and Inflammatory Gene H3K27 Acetylation

**DOI:** 10.4049/immunohorizons.2300101

**Published:** 2024-01-09

**Authors:** Kenneth K. Y. Ting, Pei Yu, Mudia Iyayi, Riley Dow, Sharon J. Hyduk, Eric Floro, Hisham Ibrahim, Saraf Karim, Chanele K. Polenz, Daniel A. Winer, Minna Woo, Jonathan Rocheleau, Jenny Jongstra-Bilen, Myron I. Cybulsky

**Affiliations:** * Toronto General Hospital Research Institute, University Health Network, Toronto, Ontario, Canada; † Department of Immunology, University of Toronto, Toronto, Ontario, Canada; ‡ Institute of Biomedical Engineering, University of Toronto, Toronto, Ontario, Canada; § Department of Laboratory Medicine and Pathobiology, University of Toronto, Toronto, Ontario, Canada; ¶ Division of Endocrinology and Metabolism, Department of Medicine, University Health Network, University of Toronto, Toronto, Ontario, Canada; ‖ Banting and Best Diabetes Centre, University of Toronto, Toronto, Ontario, Canada; # Department of Physiology, University of Toronto, Toronto, Ontario, Canada; ** Peter Munk Cardiac Centre, University Health Network, Toronto, Ontario, Canada

## Abstract

The accumulation of lipid and the formation of macrophage foam cells is a hallmark of atherosclerosis, a chronic inflammatory disease. To better understand the role of macrophage lipid accumulation in inflammation during atherogenesis, we studied early molecular events that follow the accumulation of oxidized low-density lipoprotein (oxLDL) in cultured mouse macrophages. We previously showed that oxLDL accumulation downregulates the inflammatory response in conjunction with downregulation of late-phase glycolysis. In this study, we show that within hours after LPS stimulation, macrophages with accumulated oxLDL maintain early-phase glycolysis but selectively downregulate activation of AKT2, one of three AKT isoforms. The inhibition of AKT2 activation reduced LPS-induced ATP citrate lyase activation, acetyl-CoA production, and acetylation of histone 3 lysine 27 (H3K27ac) in certain inflammatory gene promoters. In contrast to oxLDL, multiple early LPS-induced signaling pathways were inhibited in macrophages with accumulated cholesterol, including TBK1, AKT1, AKT2, MAPK, and NF-κB, and early-phase glycolysis. The selective inhibition of LPS-induced AKT2 activation was dependent on the generation of mitochondrial oxygen radicals during the accumulation of oxLDL in macrophages prior to LPS stimulation. This is consistent with increased oxidative phosphorylation, fatty acid synthesis, and oxidation pathways found by comparative transcriptomic analyses of oxLDL-loaded versus control macrophages. Our study shows a functional connection between oxLDL accumulation, inactivation of AKT2, and the inhibition of certain inflammatory genes through epigenetic changes that occur soon after LPS stimulation, independent of early-phase glycolysis.

## Introduction

Atherosclerosis is a chronic inflammatory disease that underlies ischemic conditions such as myocardial infarction and stroke. The accumulation of cholesterol-rich plaques in the intimal layer of large or medium-size arteries is a characteristic feature of atherosclerosis, and macrophage foam cells constitute a major component of these atherosclerotic plaques. In general, foam cells are formed through intracellular accumulation of modified forms of low-density lipoprotein (LDL) particles, including oxidized LDL (oxLDL) (1). oxLDL can be taken up by macrophages (Mφs) through scavenger receptors, such as CD36, Msr1, and SRA (2, 3). Following uptake, oxLDL particles are then processed in lysosomes, and esterified cholesterol is stored in lipid droplets (4). We have previously shown that the accumulation of oxLDL or cholesterol in cultured Mφs impaired their inflammatory gene expression in response to TLR agonists (5). This unexpected observation was supported by other studies including recent transcriptomics analyses of atheromas from mice and humans (6, 7) suggesting that dampening of inflammatory gene expression in foam cells is an adaptation in atherosclerosis plaques.

Past studies from the immunometabolism field have comprehensively demonstrated that metabolic reprogramming from oxidative phosphorylation to glycolysis is critical for orchestrating the inflammatory response in myeloid cells (8). Specifically, myeloid cells undergo early and late phases of glycolytic reprogramming to support their inflammatory functions (9, 10). The LPS-induced early phase of glycolysis (prior to 4 h) is dependent on early TLR4 signaling, whereas the late phase (after 4 h) is dependent on the stabilization and transcriptional activity of HIF-1α for the expression of IL-1β and other genes associated with inflammation and glycolysis (11, 12). Recently, our study showed that oxLDL accumulation suppresses late-phase glycolysis and dampens macrophage inflammatory gene expression (13). However, how oxLDL accumulation regulates the early phase of glycolysis in Mφs remains to be determined.

One of the early TLR4 signaling events is the activation of the phosphoinositide-dependent serine/threonine protein kinase AKT, also known as protein kinase B. Classical activation of AKT depends on high-affinity binding of its pleckstrin homology domain to the membrane-anchored phosphatidylinositol (3,4,5)-trisphosphate (PIP_3_), which is generated from phosphatidylinositol (4,5)-bisphosphate (PIP_2_) by class I PI3Ks (14, 15). Membrane-bound AKT is activated once it is fully phosphorylated at Thr^308^ in the kinase domain by phophoinositol-dependent kinase 1, and at Ser^473^ in the C-terminal domain by mTOR complex 2 (16, 17). Phosphatase and tensin homolog (PTEN) is a phosphatase that dephosphorylates PIP_3_ into PIP_2_, and consequently suppresses AKT activation (18). Conversely, activation of the PI3K/AKT pathway can induce PTEN ubiquitination and degradation (19). However, in the context of TLR4 signaling, AKT has been shown to be activated by TBK1/IKKε, two closely related kinases downstream of various TLRs (9, 20). LPS-induced AKT activation promotes the translocation of hexokinase II to the mitochondrial membrane, which enhances glucose retention (9). LPS-induced AKT activation also activates ATP citrate lyase (ACLY), which converts the TCA cycle metabolite citrate into acetyl-CoA, thereby promoting fatty acid synthesis and histone acetylation (10).

To date, three AKT isoforms have been identified, and as revealed by genetic deficient models, each isoform plays nonredundant roles in the progression of atherosclerosis. For instance, AKT1 deficiency in *Akt1^−/−^Apoe^−/−^* mice led to severe atherosclerosis that was attributed to reduced levels of NO and endothelial cell viability in atherosclerosis-susceptible areas (21). In contrast, hematopoietic AKT2 deficiency in *Ldlr^−/−^* mice resulted in smaller plaques with less inflammation and promoted M2 Mφ polarization, suggesting a proinflammatory function for AKT2 in Mφs (22). Finally, AKT3 deficiency in hypercholesterolemic *Apoe^−/−^* mice or in the hematopoietic compartment led to increased foam cell and lesion formation that was associated with increased modified LDL uptake and cholesterol esterification (23). In summary, the functions of AKT1 and AKT3 are atheroprotective, whereas AKT2 promotes inflammation and lesion formation.

In this study, we show that oxLDL accumulation in Mφs impaired LPS-induced activation of AKT2 and ACLY, the production of acetyl-CoA, and the acetylation of histone 3 lysine 27 (H3K27) in inflammatory gene promoters, all within 3 h of LPS stimulation. The inhibition of AKT2 activity was dependent on oxLDL-induced mitochondrial oxidative stress, and the effects of oxLDL appeared to be uncoupled from TLR4-mediated signaling and induction of early-phase glycolysis. These finding are distinct from cholesterol accumulation in Mφs, which inhibited multiple LPS-induced signaling pathways and early-phase glycolysis.

## Materials and Methods

### Mouse strains

Eight- to 12-wk-old mice were used. C57BL/6J (strain no. 000664), B6.129P2-*Lyz2^tm1(cre)Ifo^*/J (strain no. 004781), and B6.129S4-*Pten^tm1Hwu^*/J mice (strain no. 006440) were purchased from The Jackson Laboratory. *Lyz2-*Cre:*Pten^fl/fl^* mice were generated by backcrossing *Lyz2-*Cre into *Pten^fl/fl^* mice. Breeding for experiments consisted of crosses between Cre-positive and Cre-negative *Pten^fl/fl^* mice. All mice were maintained in a pathogen-free, temperature-regulated environment with a 12-h light/12-h dark cycle and were fed a normal chow diet (16 kcal % fat). All studies were performed under the approval of Animal User Protocols by the Animal Care Committee at the University Health Network according to the guidelines of the Canadian Council on Animal Care. Littermates were used for experiments (see below).

### Thioglycolate-elicited peritoneal Mφ isolation

For each experiment, one or two litters of male and female wild-type C57BL/6J mice 8–12 wk of age (up to 10 mice in total) were injected i.p. with 1 ml of 4% aged thioglycolate (Thermo Fisher Scientific, no. 211716) and peritoneal Mφs (PMφs) were harvested after 4 d by lavage with cold PBS containing 2% FBS. Cells were pooled, counted, divided into four experimental groups, and cultured (37°C, 5% CO_2_) in DMEM supplemented with 10% FBS, 2 mM l-glutamine, and 10,000 U/ml penicillin/streptomycin. Nonadherent cells were washed away and adherent PMφs were used in experiments after 18 h.

### Bone marrow–derived Mφ generation

Bone marrow–derived Mφs (BMDMφs) were obtained from Cre^+^ and Cre^−^ *Pten^fl/fl^* mice. Sex-matched littermates of up to five mice per experiment (Cre^+^ and Cre^−^) were used. Mice were euthanized in a CO_2_ chamber and bone marrow cells were isolated from leg bones. Cells were cultured (37°C, 5% CO_2_) in RPMI 1640 supplemented with 10% FBS, 2 mM l-glutamine, 10,000 U/ml penicillin/streptomycin, and 40 ng/ml M-CSF (PeproTech, no. AF-315-02) for 7 d. Cells were counted and replated for experiments. Male and female mice were used in different experiments. The results were comparable and therefore the data were combined.

### Lipid accumulation, LPS stimulation, and inhibitor studies

BMDMφs and PMφs were cultured for 24 h with human medium oxLDL (100 μg/ml, Kalen Biomedical, no. 770202) or cholesterol (50 μg/ml, Sigma-Aldrich, no. C3045), followed by ultrapure LPS stimulation (10 ng/ml, InvivoGen, no. tlrl-3pelps) for up to 6 h, or CpG (300 nM) (InvivoGen, no. tlrl-1826), Pam_3_CSK_4_ (10 ng/ml) (Tocris Bioscience, no. 4633), or bpV (HOpic) (10 µM) (Sigma-Aldrich, no. SML0884) stimulation for up to 3 h. Ethanol (0.5%) was used as a carrier control for cholesterol. For inhibitor experiments, MK-2206 (3 µM) (Cayman Chemical, no. 11593), AKT2i (3 µM) (Sigma-Aldrich, no. 124029), Mito-TEMPO hydrate (25 µM) (Cayman Chemical, no. 16621), and bpV (HOpic) (10 µM) were added 1 h prior to LPS stimulation. For assessing the accumulation of oxLDL, a mixture of DiI-oxLDL and oxLDL (10 and 90 μg/ml, respectively) were added to PMφs for 24 h. In all experiments, Mφs were first cultured for 24 h with or without oxLDL or cholesterol. The groups without oxLDL or cholesterol served as controls. The control group for cholesterol loading was cultured with media containing the same concentration of ethanol as the cholesterol group. Cells were then stimulated with LPS from 0 up to 3 h. At each time point, the groups with versus without oxLDL or cholesterol were compared and changes over time were determined.

### Immunoblotting

PMφs were cultured in 12-well plates at 2 × 10^6^ per experimental condition. Cells were lysed in ice-cold RIPA buffer (1% Nonidet P-40, 0.1% SDS, 0.5% deoxycholate in PBS, supplemented with 1 mM PMSF, 1× complete EDTA-free mini protease inhibitor cocktail [Sigma-Aldrich, no. 11873580001] and 1× PhosSTOP [Sigma-Aldrich, no. 4906845001]) for 15 min. Protein concentrations in lysates were determined by protein assay dye reagent (Bio-Rad, no. 5000006), diluted in 2× Laemmli sample buffer (Bio-Rad, no. 161-0737) with fresh 2-ME (Bio-Rad, no. 1610710), and heated at 95°C for 5 min. Samples (20 μg of protein per lane) were resolved on 8–15% SDS-PAGE gels and transferred to polyvinylidene difluoride membranes (Sigma-Aldrich, no. IPVH00010) using a wet transfer system. Membranes were blocked with 5% skim milk nonfat powder or 3% BSA (BioShop, no. ALB003) in TBST for 1 h at room temperature. Membranes were incubated with primary Abs overnight as follows: anti-actin (Sigma-Aldrich, no. A2066), anti–p-AKT (Ser^473^) (CST, no. 4060), anti–p-AKT (Thr^308^) (CST, no. 4056), AKT (CST, no. 4685), anti–p-PRAS40 (Thr^246^) (CST, no. 13175), anti-PRAS40 (CST, no. 2691), anti–p-S6K (Thr^389^) (CST, no. 9234), anti-S6K (CST, no. 9202), anti–p-S6 (Ser^235/236^) (CST, no. 4858), anti-S6 (CST, no. 2217), anti–p-4EBP1 (Ser^65^) (CST, no. 9451), anti-4EBP1 (CST, no. 9644), anti–p-GSK3α/β (Ser^21/9^) (CST, no. 9327), anti–p-GSK3α/β (CST, no. 5676), anti–p-AKT1 (Ser^473^) (CST, no. 9018), anti-AKT1 (CST, no. 2938), anti–p-AKT2 (Ser^474^) (CST, no. 8599), anti-AKT2 (CST, no. 2964), anti–p-AKT3 (Ser^472^) (Biorbyt, no. orb6790), anti-AKT3 (CST, no. 3788), anti–p-ACLY (Ser^455^) (CST, no. 4331), anti-ACLY (Abcam, no. ab40793), anti–p-TBK1 (Thr^172^) (CST, no. 5483), anti-TBK1 (CST, no. 38066), anti–p-p65 (Ser^536^) (CST, no. 3033), anti-p65 (CST, no. 8242), anti–p-ERK(Thr^202^/Tyr^204^) (CST, no. 4370), and anti-ERK (CST, no. 4695), followed by washing and incubation with HRP-conjugated anti-rabbit IgG (CST, no. 7074) (22°C, 1 h). Blots were developed using Immobilon Forte Western HRP substrate (Sigma-Aldrich, no. WBLUF0100), imaged with MicroChemi 4.2 (Bio-Rad), and analyzed with ImageJ. All phospho-blots were stripped with Western blot stripping buffer (21059X4, ThermoFisher) for 1 h at room temperature. Blots were then washed three times (5 min per wash) with 1× TBST buffer and blocked with 5% skim milk (in 1× TBST) for 30 min at room temperature. The stripped blots were then incubated overnight with Ab recognizing the corresponding total protein.

### Quantification of metabolites

To quantify acetyl-CoA, an acetyl-CoA assay kit (Sigma-Aldrich, no. MAK039A) was used. In brief, 1 × 10^6^ PMφs were plated in each well of a 24-well plate per experimental condition, after which 150 µl of acetyl-CoA assay buffer was first added per well followed by mechanical scraping of cells. Cell lysates were then used to quantify the amount of acetyl-CoA by following the manufacturer’s instructions. To quantify intracellular lactate, an l-lactate assay kit (Cayman Chemical, no. 700510) was used. In brief, 2 × 10^6^ PMφs were plated in each well of a 12-well plate per experimental condition. After treatment, cells were washed with cold PBS three times, and lactate assay buffer was added to each well followed by mechanical scraping of cells. Cell lysates were then used to quantify the amount of intracellular lactate by following the manufacturer’s instructions. To quantify extracellular lactate, an l-lactate assay kit I (Eton Bioscience, no. 1200012002) was used. In brief, 2 × 10^6^ PMφs were plated in each well of a 12-well plate per experimental condition. To reduce background, PMφs were cultured in phenol red–free Opti-MEM (Thermo Fisher Scientific, no. 11058021) during treatment. After treatment, media were filtered using 10-kDa filtered columns (Abcam, no. ab93349). Filtered media were then used to quantify the amount of extracellular lactate by following the manufacturer’s instructions.

### Extracellular acidification rate measurement

PMφs (2.5 × 10^5^) were cultured in XFe24 well plates (Agilent Technologies, no. 102342-100). For glycolysis stress tests, cells were stimulated for 3 h with LPS, washed three times with Seahorse XF DMEM (Agilent Technologies, 103334-100) supplemented with 1 mM glutamine and 2 mM pyruvate, and incubated (37°C, 0% CO_2_) for 30 min prior to the test. During the test, glucose (Agilent Technologies, no. 103577-100), oligomycin A (Cayman Chemical, no. 11342), and 2-deoxyglucose (Sigma-Aldrich, no. D8375) were added by the XFe24 Seahorse analyzer (final concentrations were 25 mM, 2 μM, and 25 mM, respectively).

### Glucose uptake, reactive oxygen species, and lysosomal pH measurement

PMφs (3 × 10^6^) were cultured in a 35-mm petri dish, with 14-mm microwells (MatTek, no. P35G-1.5-14-C). After treatment, cells were washed three times with prewarmed HBSS (Wisent, no. 311-513-CL). For the glucose uptake assay, cells were cultured for 1 h at 37°C, 5% CO_2_ in DMEM without glucose (Thermo Fisher Scientific, no. 11966025) supplemented with 10% FBS, 2-deoxy-2-[(7-nitro-2,1,3-benzoxadiazol-4-yl)amino]-d-glucose (2-NBDG, Cayman Chemical, no. 11046) (100 μg/ml) and 32.4 μM Hoechst nuclear staining reagent (Thermo Fisher Scientific, no. H3570). For the reactive oxygen species (ROS) assay, cells were cultured for 1 h at 37°C, 5% CO_2_ in HBSS, supplemented with MitoSOX Red (5 μM, Thermo Fisher Scientific, no. M36008) and Hoechst. For lysosomal pH measurement, cells were cultured for 1 h at 37°C, 5% CO_2_ in HBSS, supplemented LysoTracker Red (50 nM, Thermo Fisher Scientific, no. L7528). Cells were then washed three times with prewarmed HBSS. For glucose uptake assay, cells were live imaged with an Olympus FluoView 1000 laser scanning confocal microscope (Olympus America).

### RNA isolation and real-time PCR

Total RNA was isolated with an E.Z.N.A. total RNA kit I (Omega Bio-tek, no. R6834-01), and reverse transcription reactions were performed with a high-capacity cDNA reverse transcription kit (Thermo Fisher Scientific, no. 4368814) according to the manufacturers’ protocols. Real-time quantitative PCR (qPCR) was then performed using a Roche LightCycler 480 with Luna universal qPCR master mix (New England Biolabs, no. M3003E). Quantification of mRNA was performed by using primers that span over two adjacent exons, quantified using the comparative standard curve method, and normalized to hypoxanthine phosphoribosyltransferase (*Hprt*) as the housekeeping gene. Primer sequences used for qPCR are as follows: *Il1b*, forward, 5′-AGTTGACGGACCCCAAAAGA-3′, reverse, 5′-TGCTGCTGCGAGATTTGAAG-3′; *Il6*, forward, 5′-CTCCCAACAGACCTGTCTATACCA-3′, reverse, 5′-TGCCATTGCACAACTCTTTTCT-3′; *Il12b*, forward, 5′-AAGTGGGCATGTGTTCC-3′, reverse, 5′-TCTTCCTTAATGTCTTCCACTT-3′; *Il15*, forward, 5′-GTAGGTCTCCCTAAAACAGAGGC-3′, reverse, 5′-TCCAGGAGAAAGCAGTTCATTGC-3′; *Il18*, forward, 5′-ACAGGCCTGACATCTTCTGC-3′, reverse, 5′-CCTTGAAGTTGACGCAAGAGT-3′; *Il27*, forward, 5′-TCTCGATTGCCAGGAGTGAACC-3′, reverse, 5′-AGTGTGGTAGCGAGGAAGCAGA-3′; *Ccl3*, forward, 5′-CCCAGCCAGGTGTCATTT-3′, reverse, 5′-AGTTCCAGGTCAGTGATGTATTC-3′; *Ccl4*, forward, 5′-ACCCTCCCACTTCCTGCTGTTT-3′, reverse, 5′-CTGTCTGCCTCTTTTGGTCAGG-3′; *Ccl5*, forward, 5′-CCTGCTGCTTTGCCTACCTCTC-3′, reverse, 5′-ACACACTTGGCGGTTCCTTCGA-3′; *Ccl8*, forward, 5′-GGGTGCTGAAAAGCTACGAGAG-3′, reverse, 5′-GGATCTCCATGTACTCACTGACC-3′; *Ccl9*, forward, 5′-TCCAGAGCAGTCTGAAGGCACA-3′, reverse, 5′-CCGTGAGTTATAGGACAGGCAG-3′; *Ccl22*, forward, 5′-GTGGAAGACAGTATCTGCTGCC-3′, reverse, 5′-AGGCTTGCGGCAGGATTTTGAG-3′; *Tnfa*, forward, 5′-GTAGCCCACGTCGTAGCAAAC-3′, reverse, 5′-GCACCACTAGTTGGTTGTCTTTGA-3′; *Pten*, forward, 5′-TGAGTTCCCTCAGCCATTGCCT-3′, reverse, 5′-GAGGTTTCCTCTGGTCCTGGTA-3′; *Cd36*, forward, 5′-GCCAAGCTATTGCGACATGA-3′, reverse, 5′-AAAAGAATCTCAATGTCCGAGACTTT-3′; *Lipa*, forward, 5′-ATCCTGGTGAGGAACACTCGGT-3′, reverse, 5′-TAGAATCTGCCAGCAAGCCGTG-3′; *Cpt1a*, forward, 5′-GGCATAAACGCAGAGCATTCCTG-3′, reverse, 5′-CAGTGTCCATCCTCTGAGTAGC-3′; *Acaa2*, forward, 5′-TCTGCTGGCAAAGTTCCACCTG-3′, reverse, 5′-ACAGAGCCTGTTGAGGGTAAGG-3′; *Hprt*, forward, 5′-CAAGCTTGCTGGTGAAAAGGA-3′, reverse, 5′-TGAAGTACTCATTATAGTCAAGGGCATATC-3′.

### H3K27 acetylation and chromatin immunoprecipitation–PCR

BMDMφs and PMφs (4 × 10^6^) were plated in each well per condition in a six-well plate. Following oxLDL accumulation and LPS treatment, Mφs were cross-linked with 1% formaldehyde in PBS at room temperature for 15 min, then quenched with glycine at a final concentration of 0.125 M for another 15 min. After cross-linking, Mφs were lysed with cytoplasmic lysis buffer (1 ml per well) (10 mM HEPES/KOH [pH 7.9], 85 mM KCl, 1 mM EDTA [pH 8.0], 0.5% Nonidet P-40, 1 mM PMSF, and 1× protease inhibitor) for 10 min on ice, then scraped and pelleted at 700 × *g* for 5 min at 4°C. The pellet was resuspended with nuclear lysis buffer (10 mM Tris/HCl, 100 mM NaCl, 10 mM EDTA, 0.5 mM EGTA, 0.1% deoxycholate, 0.5% SDS, 1× protease inhibitor, 1 mM PMSF) and then sonicated for 40 cycles at 4°C (30 s on, 30 s off). Dilution buffer (20 mM Tris/HCl, 100 mM NaCl, 2 mM EDTA, 0.5% Trion X-100, 1× protease inhibitor) was then added to the sonicated lysates (1 in 2.5 dilution) and 1% of the diluted lysates were saved as input. The diluted lysates were then incubated overnight with protein A Dynabeads (Thermo Fisher Scientific, 10001D) that were previously incubated with 2 µg of anti-histone H3 (acetyl K27) Ab (Abcam, Ab4729) at 4°C for 3 h. On the next day, bead complexes were washed five times with buffer 1 (20 mM Tris/HCl [pH 7.4], 150 mM NaCl, 0.1% SDS, 1% Triton X-100, 2 mM EDTA), four times with buffer 2 (10 mM Tris/HCl [pH 7.4], 250 mM LiCl, 1% Nonidet P-40, 0.7% deoxycholate, 1 mM EDTA [pH 8.0]), twice with buffer 3 (0.2% of Triton X-100 in 10 mM Tris-HCl and 1 mM EDTA), and twice with buffer 4 (50 mM NaCl in 10 mM Tris-HCl and 1 mM EDTA). The bound DNA was eluted from bead complexes with elution buffer (2% SDS in 10 mM Tris-HCl and 1 mM EDTA) at room temperature shaking for 30 min. After elution, 0.283 M NaCl was added to both input and elution to reverse cross-link overnight at 65°C. On the next day, 67 µg of DNase-free RNase (Thermo Fisher Scientific, no. EN0531) was added to each sample and incubated at 37°C for 1.5 h, after which 100 µg of proteinase K was added to each sample and incubated at 55°C for 1 h. All of the samples were then purified with a PCR purification kit (Qiagen, no. 28104) for subsequent PCR assays. Primers used for chromatin immunoprecipitation (ChIP)–PCR assay are as follows: *Il1a*, forward, 5′-GTGTGGGGAGACCTTGATCC-3′, reverse, 5′-ATCTTCCAGCTTCAGGGTGC-3′; *Il1b*, forward, 5′-GAGTGTCAGCTCCAAGTCCC-3′, reverse, 5′-GACAACCACTCCTCTCAGGC-3′; *Il6*, forward, 5′-CCACTGGGGAGAATGCAGA-3′, reverse, 5′-GGAGTTGCCAGGTGGGTAAA-3′; *Ccl5*, forward, 5′-AGGCTCCAGCGTTTACTTCC-3′, reverse, 5′-CTGACACTGAGGGCTGATGG-3′; *Hprt*, forward, 5′-TCTGGCTAGATGCCCCATGA-3′, reverse, 5′-CATTGCCACCTTCCTGGTCC-3′.

### Bulk RNA sequencing library preparation

Total RNA was extracted from 2 × 10^6^ PMφs using an RNeasy mini kit (Qiagen, no. 74104). To eliminate genomic DNA, 20 U of Roche RNase-free DNase I (Roche, no. 4716728001) was added in the eluant (100 µl), followed by 20 min of incubation at 37°C. Samples were then passed through RNeasy columns. Total RNA was quantified by a NanoDrop ND-1000 spectrophotometer, and 750 ng was used to enrich mRNA using an NEBNext poly(A) mRNA magnetic isolation module (New England Biolabs, no. E7490). Library construction followed immediately using an NEBNext Ultra II directional RNA library prep kit (New England Biolabs, no. E7760) and NEBNext multiple oligonucleotides for Illumina (New England Biolabs, nos. E7335, E7500, E7710, E7730). Libraries were PCR amplified for 10 cycles, quantified by MiSeq Nano v2, and sequenced on an Illumina NovaSeq S1 flow cell with a 150-bp paired-end run to obtain ∼30 million reads per sample. The complete RNA sequencing (RNA-seq) dataset has been deposited to the GEO repository (GSE239696; https://www.ncbi.nlm.nih.gov/geo/query/acc.cgi?acc=GSE239696).

### Bulk RNA-seq data acquisition and processing

The adaptor sequences were removed using Trimmomatic (v0.32; recommended parameters). The reads were aligned to the mouse genome reference assembly mm10 (GRCm38) using STAR (v2.5.1b; default parameters). The quality metrics were done using MultiQC (v1.3). To count exonic reads we used featureCounts (http://subread.sourceforge.net/, v1.5.0) and annotated them for mouse genes using GENCODE vM4.

### Bulk RNA-seq differential expression and pathway analysis

The DESeq2 R/Bioconductor package was used to perform hierarchical cluster analysis, principal component analysis, and to determine differential expression between Mφs with and without oxLDL. Differential expression was assessed using the Wald test with a Benjamini–Hochberg correction; genes with an adjusted *p* value of <0.1 were considered significantly differentially expressed. EnhancedVolcano (version 1.11.3) was used to create volcano plots. Genes were preranked using the DESeq2 output (−log_10_(*p* value) × sign(log fold change)) for enrichment analysis. Pathway enrichment was performed using gene set enrichment analysis software (version 4.1.0) from the Broad Institute (https://software.broadinstitute.org/cancer/software/gsea/wiki/index.php/Main_Page).

### Statistical analysis

All of the statistical details of experiments can be found in the figure legends. In brief, all figures show pooled data from independent experiments. All experiments were repeated at least three times. The number of biological replicates is listed as the *n* value. Statistical analyses were performed using Prism software (9.2.0) unless otherwise specified in the figure legends.

## Results

### Both oxLDL and cholesterol accumulation in PMφs impair LPS-induced AKT signaling, whereas early glycolysis is inhibited only by cholesterol

**FIGURE 1.**
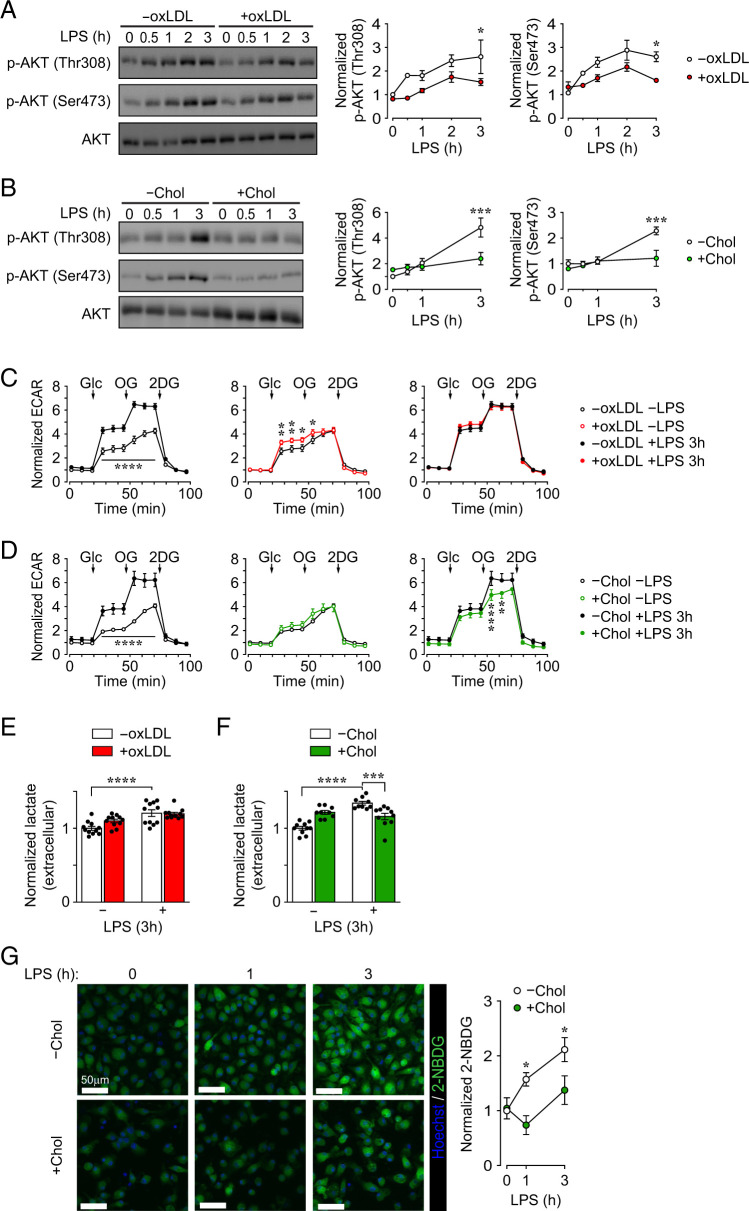
Both oxLDL and cholesterol accumulation in PMφs impair LPS-induced AKT signaling, whereas early glycolysis is inhibited only by cholesterol. (**A** and **B**) Effect of oxLDL or cholesterol (Chol) accumulation on LPS-induced AKT activation. Representative immunoblots and quantification of p-AKT (Thr^308^), p-AKT (Ser^473^), and total AKT is shown in an LPS time course experiment using PMφs with or without accumulated oxLDL (A, *n* = 4) or Chol (B, *n* = 4–6). For each time point, p-AKT values were normalized to the corresponding total AKT value and the 0 h LPS time point of the without (−)oxLDL/Chol groups (assigned a value of 1). (**C** and **D**) Seahorse analysis of PMϕs with or without LPS stimulation (3 h) and accumulation of oxLDL (C, *n* = 11–14) or Chol (D, *n* = 6–9). ECAR values were normalized to baseline of the −oxLDL/Chol −LPS groups (assigned a value of 1). The addition of glucose (Glc), oligomycin (OG), and 2-deoxyglucose (2DG) is indicated in the glycolysis stress tests. (**E** and **F**) Extracellular lactate in cell culture media. PMϕs with or without accumulated oxLDL (E, *n* = 11) or Chol (F, *n* = 9–10) were treated with LPS for 0 or 3 h. Values are normalized to −oxLDL/Chol −LPS groups (assigned a value of 1). (**G**) Representative images and quantification of 2-NBDG uptake (green) by LPS-stimulated (0–3 h) PMφs with and without Chol accumulation. Values are normalized to −Chol, 0 h LPS time point (assigned a value of 1, *n* = 10–14). The mean ± SEM is plotted in all graphs. Significant differences were determined by a two-way ANOVA and a Bonferroni post hoc test (A–D and G) or an unpaired Student *t* test (E and F). *p < 0.05, **p < 0.01, ***p < 0.001, ****p < 0.0001. +, with; −, without.

Prior to investigating the effects of oxLDL and cholesterol accumulation on LPS-induced AKT signaling and early glycolytic reprogramming in PMφs, we confirmed that LPS stimulation of PMφs activates AKT and that inhibition of AKT with MK-2206, an allosteric pan-AKT inhibitor, reduces the uptake of 2-NBDG, a fluorescent glucose analog (Supplemental Fig. 1A). Having established that LPS signaling induces AKT-dependent glucose uptake, we investigated AKT activation in PMφs with accumulated oxLDL or cholesterol. We observed diminished phosphorylation of AKT residues Ser^473^ and Thr^308^ (Fig. 1A, 1B), although in some experiments, inhibition of AKT phosphorylation was observed at earlier time points. These observations demonstrate that LPS-induced AKT activation is suppressed in PMφs with accumulated oxLDL or cholesterol. In support of this conclusion, the phosphorylation of known AKT targets, such as PRAS40 and GSK3α/β, was also suppressed in PMφs cultured with oxLDL or cholesterol (Supplemental Fig. 1B, 1C).

We next used the Seahorse bioanalyzer to measure the extracellular acidification rate (ECAR) and evaluate early glycolysis. Glycolysis stress tests were performed using control or lipid-loaded PMϕs that were unstimulated or stimulated for 3 h with LPS. As expected, LPS stimulation of control PMφs (without accumulated lipid) increased the ECAR upon the addition of glucose and oligomycin (Fig. 1C, 1D, left panels), consistent with an early glycolytic response. When PMϕs were not stimulated with LPS, the ECAR was elevated relative to controls when PMϕs with accumulated oxLDL, but not cholesterol, were assayed (Fig. 1C, 1D, middle panels). Three hours after LPS stimulation, the ECAR was reduced relative to control when PMϕs with accumulated cholesterol were assayed (Fig. 1D, right panel), but it was comparable to control when PMϕs with accumulated oxLDL were assayed (Fig. 1C, right panel). Consistent with the ECAR, extracellular lactate, a metabolite generated by glycolysis, increased 3 h after LPS stimulation in control PMφs (Fig. 1E, 1F). Notably, extracellular lactate was comparable when PMφs with accumulated oxLDL were stimulated with LPS for 3 h (Fig. 1E), but it was significantly reduced in cultures of PMφs with accumulated cholesterol (Fig. 1F), consistent with the Seahorse data. Intracellular levels of lactate in PMφs with accumulated cholesterol followed a similar pattern to extracellular levels (Supplemental Fig. 1D). The Seahorse and lactate data were also supported by reduced accumulation of the glucose analog 2-NBDG following LPS stimulation of PMφs with accumulated cholesterol (Fig. 1G). In contrast to the 3 h time point, extracellular lactate was reduced when PMφs with accumulated oxLDL were stimulated with LPS for 6 h (Supplemental Fig. 1E), consistent with our previous study that focused on HIF-1α–dependent glycolysis (13). Collectively, we demonstrated that lipid accumulation in PMφs impairs LPS-induced AKT signaling; however, LPS-induced early glycolysis remains intact in PMφs with accumulated oxLDL but is suppressed in cells with accumulated cholesterol.

### oxLDL accumulation in PMφs selectively inhibits LPS-induced AKT2 and ACLY activation

**FIGURE 2.**
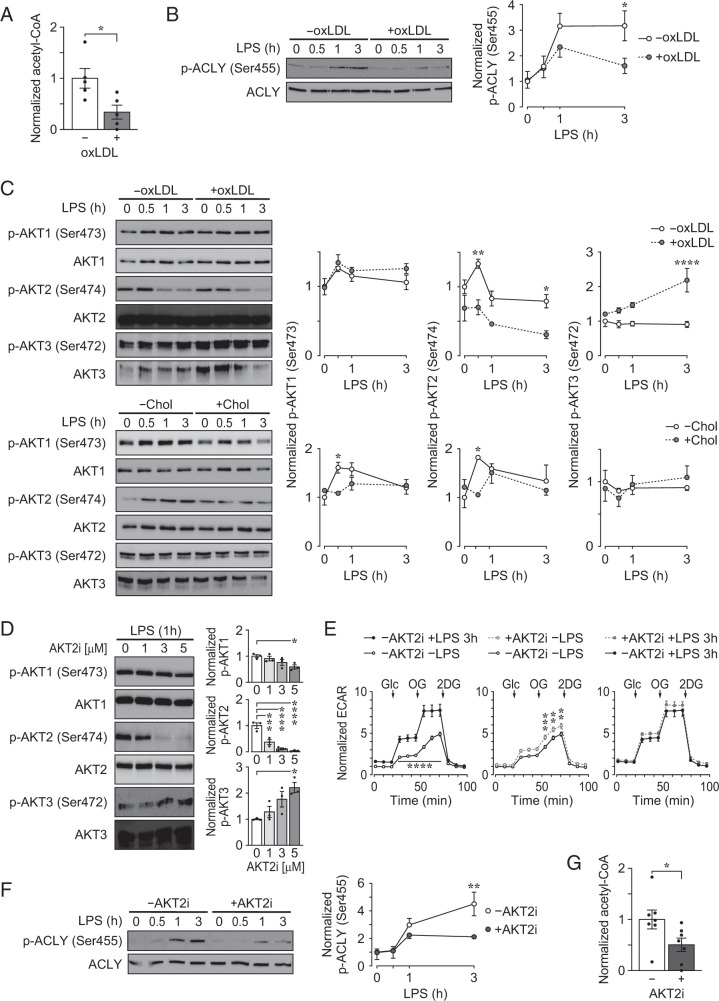
oxLDL accumulation in PMφs selectively inhibits LPS-induced AKT2 activation and reduces activation of ACLY and intracellular acetyl-CoA, and selective inhibition of AKT2 reproduces the effects of oxLDL accumulation on glycolysis, ACLY activation, and acetyl-CoA. (**A**) Intracellular acetyl-CoA in LPS-stimulated (3 h) PMφs with or without oxLDL accumulation. The data are normalized to the without (−)oxLDL group (assigned a value of 1, *n* = 5). (**B**) Effect of oxLDL accumulation on LPS-induced activation of ACLY. Representative immunoblots and quantification of p-ACLY (Ser^455^) and total ACLY in a LPS time course (0–3 h). PMφs with or without oxLDL accumulation were used. For each time point, p-ACLY values were normalized to corresponding total ACLY and the 0 h LPS time point in the −oxLDL group (assigned a value of 1, *n* = 5). (**C**) Effect of oxLDL or cholesterol (Chol) accumulation on LPS-induced activation of AKT isoforms. Representative immunoblots and quantification of p-AKT1 (Ser^473^), total AKT1, p-AKT2 (Ser^474^), total AKT2, p-AKT3 (Ser^472^), and total AKT3 in a LPS time course (0–3 h). PMφs with or without oxLDL (*n* = 3–4) or Chol (*n* = 3) accumulation were used. Values were normalized analogous to (B). (**D**) Immunoblot analysis of AKT isoform activation in LPS-stimulated PMφs treated with increasing concentration of AKT2i 1 h prior to LPS. Values were normalized analogous to (B) (*n* = 3). (**E**) Effect of AKT2i (3 µM) on LPS-induced glycolysis. Seahorse analysis of PMφs with or without LPS stimulation (3 h) and treatment with AKT2i (*n* = 10–14). ECAR values were normalized to baseline of the −AKT2i −LPS group, assigned a value of 1. The addition of glucose (Glc), oligomycin (OG), and 2-deoxyglucose (2DG) is indicated in the glycolysis stress tests. (**F** and **G**) Effect of AKT2i (3 µM) on LPS-induced activation of ACLY and intracellular acetyl-CoA. PMφs with or without AKT2i treatment were used. (F) Representative immunoblots and quantification of p-ACLY (Ser^455^) and total ACLY in a LPS time course (0–3 h). Values were normalized analogous to (B) (*n* = 3). (G) Quantification of intracellular acetyl-CoA at 3 h after LPS stimulation). The data are normalized to the −AKT2i group (assigned a value of 1, *n* = 8). The mean ± SEM is plotted in all graphs. Significant differences were determined by an unpaired Student *t* test (A and G), or a two-way ANOVA (B, C, E, and F) or one-way ANOVA (D) and a Bonferroni post hoc test. *p < 0.05, **p < 0.01, ***p < 0.001, ****p < 0.0001. +, with; −, without.

Similar to lactate, acetyl-CoA is a product of glycolysis. We determined acetyl-CoA levels at 3 h after LPS stimulation and found reduced abundance in PMφs with accumulated oxLDL (Fig. 2A). This result suggests that a pathway for acetyl-CoA generation other than glycolysis is inhibited in PMφs with accumulated oxLDL. One possibility is ACLY, an enzyme that functions in de novo lipogenesis by converting citrate to acetyl-CoA (24). We thus evaluated an established marker of ACLY activation—the phosphorylation of Ser^455^, which is a residue that is phosphorylated by AKT (25). Following LPS stimulation, ACLY Ser^455^ phosphorylation was significantly lower in PMφs with accumulated oxLDL (Fig. 2B). This implies that the inhibition of AKT signaling in cells with accumulated oxLDL inhibits ACLY activation and production of acetyl-CoA.

To gain further insights into differences in PMφ biology in the setting of oxLDL versus cholesterol accumulation, we evaluated the activation of the three AKT isoforms. LPS stimulation of control PMφs (without lipid accumulation) increased the activation of AKT1 (phosphorylation of Ser^473^) and AKT2 (phosphorylation of Ser^474^) in a time-dependent manner, but not of AKT3 (phosphorylation of Ser^472^) (Fig. 2C). In PMφs with accumulated oxLDL, AKT1 and AKT3 activation was comparable to control, but AKT2 activation was significantly reduced (Fig. 2C). In contrast, cholesterol accumulation in PMφs significantly reduced both AKT1 and AKT2 activation but not AKT3 (Fig. 2C). oxLDL accumulation selectively inhibited AKT2 activation in the RAW264.7 murine Mφ cell line, although the overall kinetics of AKT activation after LPS stimulation were distinct in these cells (Supplemental Fig. 2A). AKT2 activation in PMφs induced by other TLR agonists, including CpG-ODN (TLR9 agonist) and Pam_3_CSK_4_ (TLR1/2 agonist), was also selectively inhibited by oxLDL accumulation (Supplemental Fig. 2B).

### AKT2 inhibition reduces LPS-induced ACLY activation and acetyl-CoA, but not early glycolysis

The selective inhibitory effect of oxLDL accumulation on AKT2 activation is of interest due to its established role in promoting inflammation in Mφs (22). A relatively specific AKT2 inhibitor was employed to explore the potential role of AKT2 in inducing the early glycolytic response and ACLY activation in LPS-stimulated PMφs. Initially, we determined concentrations that selectively inhibited AKT2 activation in PMφs 1 h after LPS stimulation (Fig. 2D). We then performed glycolysis stress tests using the Seahorse bioanalyzer to assess whether AKT2 regulates early glycolysis 3 h after LPS stimulation. We found that the AKT2 inhibitor (used at 3 µM) increased the ECAR when added to unstimulated PMφs and did not reduce the ECAR when added to LPS-stimulated PMφs (Fig. 2E). However, the AKT2 inhibitor reduced LPS-induced ACLY activation in a time-dependent manner (Fig. 2F) and reduced the abundance of acetyl-CoA (Fig. 2G). This phenotype closely resembled PMφs with accumulated oxLDL (Figs. 1C, 2A, 2B).

### oxLDL accumulation selectively and irreversibly blocks AKT2 activation, whereas cholesterol accumulation inhibits LPS-induced TBK1 activation

**FIGURE 3.**
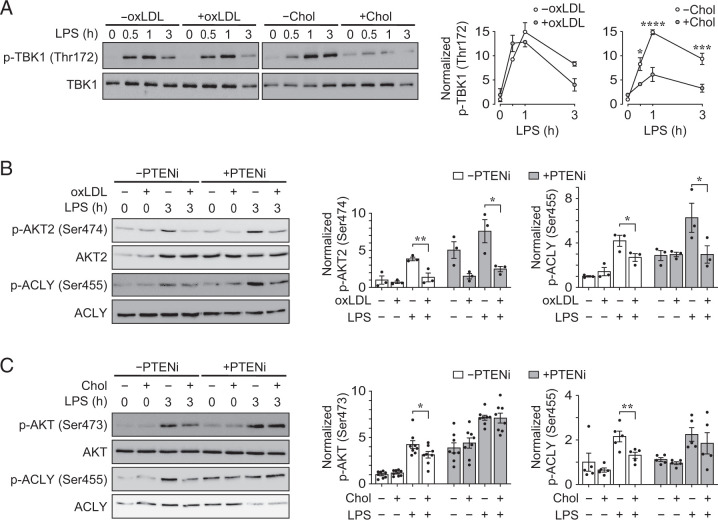
In contrast to the effects of cholesterol, oxLDL accumulation does not inhibit LPS-induced TBK1 activation, yet AKT2 and ACLY activation remain inhibited even when the localization of AKT2 to the plasma membrane is enhanced by inhibition of PTEN. (**A**) Effect of oxLDL or cholesterol (Chol) accumulation on LPS-induced activation of TBK1. Representative immunoblots and quantification of p-TBK1 (Thr^172^) and total TBK1 in a LPS time course (0–3 h). PMφs with or without oxLDL (*n* = 4) or Chol (*n* = 3) accumulation were used. For each time point, p-TBK1 values are normalized to corresponding total TBK1 and the 0 h LPS time point in the without (−)oxLDL/Chol groups (assigned a value of 1). (**B** and **C**) Effects of PTEN inhibitor (PTENi) on LPS-induced activation of AKT in PMφs with accumulated oxLDL or Chol. Representative immunoblots and quantification of p-AKT2 (Ser^474^) and total AKT2 (B), p-AKT (Ser^473^) and total AKT (C), and p-ACLY (Ser^455^) and total ACLY (B and C) in PMφs with/without (±)oxLDL accumulation (B, *n* = 3), ±Chol accumulation (C, *n* = 5–8), ±PTENi and ±LPS stimulation (3 h) are as indicated. The mean ± SEM is plotted in all graphs. Significant differences were determined by a two-way ANOVA (A) or one-way ANOVA (B and C) and a Bonferroni post hoc test. *p < 0.05, **p < 0.01, ***p < 0.001, ****p < 0.0001. +, with; −, without.

Recent studies have shown that TBK1/IKKε activate AKT and regulate AKT-dependent glycolytic reprogramming upon LPS stimulation of myeloid cells (9). We therefore evaluated the phosphorylation of TBK1 on Thr^172^ in LPS-stimulated PMφs and found that LPS-induced TBK1 phosphorylation was comparable in cells with accumulated oxLDL; however, it was reduced in cells with accumulated cholesterol (Fig. 3A). These data suggest that signaling upstream of AKT is inhibited in PMφs with accumulated cholesterol but not in cells with accumulated oxLDL. Nevertheless, oxLDL selectively inhibited AKT2 activation (Fig. 2C).

The activation of AKT isoforms requires localization to the plasma membrane through binding of an AKT pleckstrin homology domain to PIP_3_. Plasma membrane localization of AKT can be increased by inhibiting PTEN, which converts PIP_3_ to PIP_2_ by dephosphorylating the 3′ phosphate. We obtained bisperoxovanadium (HOpic), a potent PTEN inhibitor (PTENi) and performed a dose-response experiment to determine that 10 µM is the optimal concentration (Supplemental Fig. 3A). After PMφs accumulated oxLDL or cholesterol, cells were treated with PTENi for 1 h prior to LPS stimulation. In PMφs with accumulated oxLDL, LPS-induced activation of both AKT2 and ACLY remained suppressed when cells were treated with PTENi (Fig. 3B). Treatment with PTENi rescued LPS-induced activation of both AKT and ACLY, which was inhibited by cholesterol accumulation (Fig. 3C).

Collectively, we demonstrated that the accumulation of oxLDL in PMφs does not inhibit LPS-induced TBK1 activation; nevertheless, AKT2 and ACLY activation is suppressed and cannot be rescued by inhibition of PTEN. This suggests that the impaired activation of AKT2 is unlikely due to defective upstream kinase signaling cascades, as oxLDL in PMφs does not block most TLR4-induced signaling pathways, including TBK1 (Fig. 3A), MAPK, and NF-κB (5). In contrast, the accumulation of cholesterol in PMφs inhibits LPS-induced TBK1 activation (Fig. 3A), which accounts for suppressed AKT and ACLY activation, and the restoration of their inhibition by inhibiting PTEN (Fig. 3C). This suggests that cholesterol loading interferes with phosphorylation cascades upstream of AKT2.

### Mitochondrial oxidative stress in response to oxLDL accumulation inactivates AKT2

In the absence of LPS stimulation, treatment of control PMφs with the PTENi increased the activation of AKT1 and AKT2 but not AKT3 (Supplemental Fig. 3B). PTENi-induced activation of AKT2, but not AKT1, was inhibited in PMφs with accumulated oxLDL (Supplemental Fig. 3B). These observations together with the differential effects of PTENi in LPS-stimulated cells with accumulated oxLDL versus cholesterol (Fig. 3B, 3C) suggested the possibility that AKT2 is intrinsically deactivated by oxLDL loading.

**FIGURE 4.**
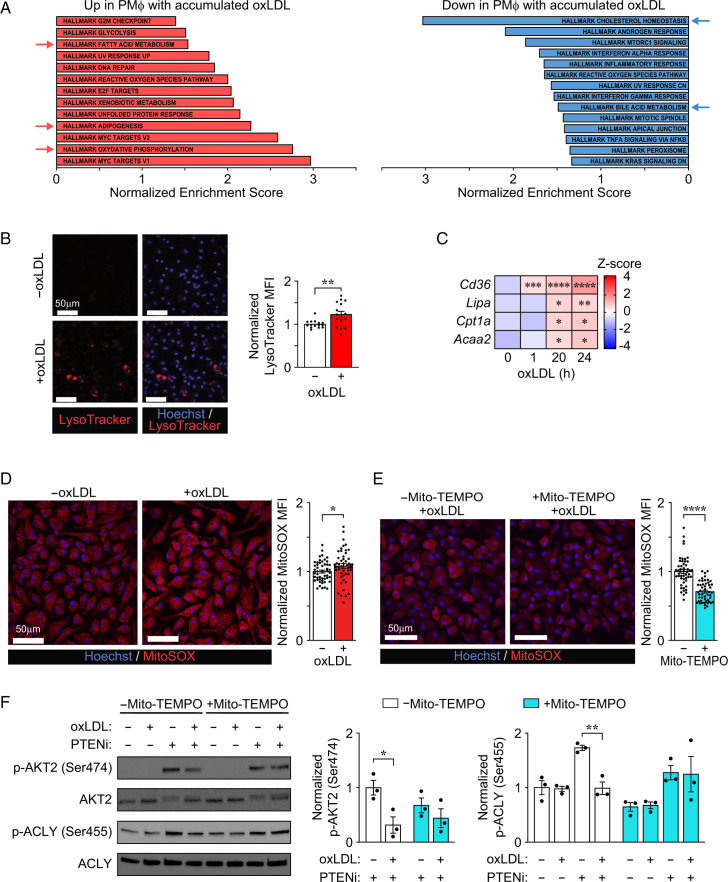
The accumulation of oxLDL in PMφs generates mitochondrial oxidative stress, which inactivates AKT2. (**A**) Hallmark pathway analysis of significantly upregulated and downregulated genes (*p* < 0.01) in oxLDL versus control PMφs. Red arrows indicated fatty acid metabolism-related pathways. Blue arrows indicate cholesterol metabolism-related pathways. (**B**) LysoTracker staining in PMφs with or without accumulated oxLDL. Representative fluorescence microscope images and quantification of LysoTracker mean fluorescence intensity (MFI) (*n* = 15 fields, >3 independent experiments). (**C**) Heat map of fatty acid oxidation gene mRNA expression assessed by qPCR in PMφs during sequential time points of oxLDL accumulation (*n* = 3). (**D** and **E**) MitoSOX staining of PMφs. (D) Staining at 0 or 18 h after accumulation of oxLDL. (E) Staining of cells with or without Mito-TEMPO (25 µM) treatment 1 h prior to culture with oxLDL for 18 h. Shown are representative fluorescence microscope images and quantification of MitoSOX MFI (*n* = 50 fields, >3 independent experiments). (**F**) Effect of mitochondrial ROS suppression on the activation of AKT2 and ACLY in response to PTEN inhibition in PMφs with or without oxLDL accumulation. Representative immunoblots and quantification of p-AKT2 (Ser^474^), total AKT2, p-ACLY (Ser^455^), and total ACLY in PMφs with or without Mito-TEMPO (added 1 h prior to oxLDL), oxLDL accumulation (24-h), and PTENi (3-h treatment after culture with oxLDL) are as indicated. The p-AKT2 and p-ACLY values were normalized to the corresponding total AKT2 or total ACLY and the without (−)Mito-TEMPO −oxLDL groups (assigned a value of 1, *n* = 3). The mean ± SEM is plotted in all graphs. Significant differences were determined by an unpaired Student *t *test (B, D, and E) or a one-way ANOVA (C) or two-way ANOVA (F). *p < 0.05, **p < 0.01, ***p < 0.001, ****p < 0.0001. +, with; −, without.

Past research has shown that the activation of AKT2 can be regulated by oxidative stress (26, 27). Because we have previously showed that oxLDL accumulation in PMφs induces oxidative stress (13), this suggests the possibility that oxLDL-induced ROS deactivate AKT2. To test this, we first explored the potential source of ROS by comparing the transcriptomes of PMφs with or without oxLDL accumulation with RNA-seq. Principal component analysis showed that oxLDL did not induce wide-scale transcriptome alterations, yet the expression of certain metabolic genes such as CD36 was induced significantly (Supplemental Fig. 3C, 3D). Hallmark pathway analyses revealed that oxLDL accumulation significantly altered intracellular metabolic processes, including upregulation of oxidative phosphorylation, adipogenesis, and fatty acid metabolism and downregulation of cholesterol homeostasis and bile acid metabolism (Fig. 4A). Of particular interest is the increase in fatty acid oxidation, as it has been associated with impaired inflammatory responses in Mφs (28). The breakdown of lipid droplets in lysosomes releases free fatty acids. We stained PMφs with LysoTracker and found evidence of activated lysosomal function upon accumulation of oxLDL (Fig. 4B). We also measured by qPCR increased expression of genes that regulate fatty acid oxidation, such as *Cd36*, *Lipa*, *Cpt1a*, and *Acaa2* (Fig. 4C). Because fatty acid oxidation can fuel the TCA cycle and generate energy in the mitochondria, we hypothesized that oxLDL-induced ROS are generated in mitochondria. Indeed, staining with MitoSOX showed increased mitochondrial ROS in PMφs with accumulated oxLDL (Fig. 4D). Therefore, Mito-TEMPO, a mitochondrial-targeted antioxidant, was added 1 h prior to culture of PMφs with oxLDL. This treatment with Mito-TEMPO did not alter oxLDL accumulation in PMφs (Supplemental Fig. 4A) but it reduced mitochondrial ROS in PMφs with accumulated oxLDL (Fig. 4E). Mito-TEMPO was then used to determine whether mitochondrial-derived ROS underlie the deactivation of AKT2 in PMφs with accumulated oxLDL. Unfortunately, Mito-TEMPO inhibited LPS-induced activation of AKT2 (Supplemental Fig. 4B); therefore, we used PTENi to activate AKT2 and AKT1 (Supplemental Fig. 3B) and found that PTENi-induced activation of AKT2 and ACLY was restored by Mito-TEMPO in PMφs with accumulated oxLDL (Fig. 4F). This finding is consistent with the possibility that generation of mitochondrial ROS in response to oxLDL accumulation modifies and inactivates AKT2.

### oxLDL accumulation in PMφs suppresses inflammatory gene expression 3 h after LPS stimulation, and cholesterol accumulation inhibits LPS-induced signaling

**FIGURE 5.**
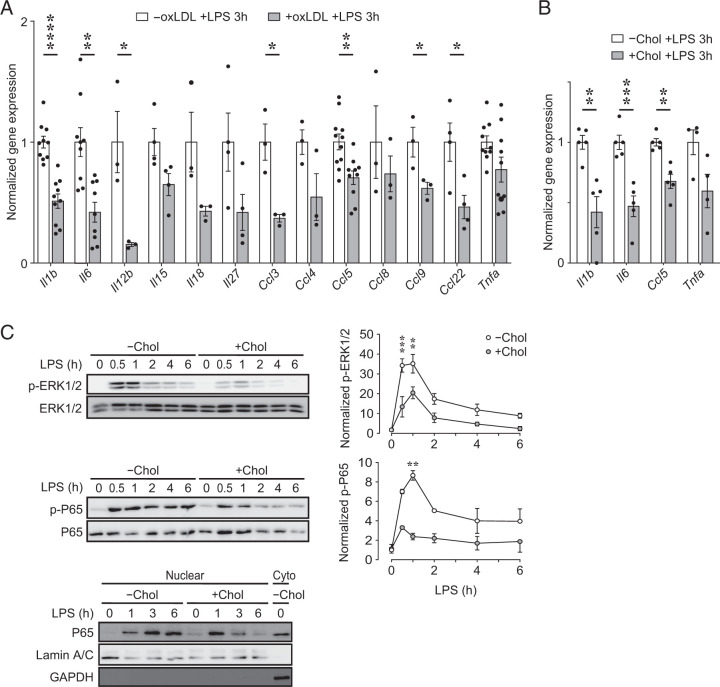
oxLDL accumulation in PMφs suppresses inflammatory gene expression at 3 h after LPS stimulation, and cholesterol accumulation inhibits LPS-induced signaling. (**A** and **B**) Analysis of proinflammatory gene mRNA expression by qPCR in LPS-stimulated (3 h) PMφs with or without accumulated oxLDL (A, *n* = 3–11) or cholesterol (Chol) (B, *n* = 4–5). For each gene the data were normalized to cells without oxLDL or cholesterol (assigned a value of 1). (**C**) Representative immunoblots and quantification of p-ERK1/2 (Thr^202^/Tyr^204^), total ERK, p-p65 (Ser^536^), and total p65 in PMφs with or without Chol accumulation after LPS stimulation (0–6 h). Values were normalized to the 0 h LPS time point in PMφs without Chol (*n* = 3). A representative immunoblot of nuclear and cytoplasmic (Cyto) fractions shows the post-LPS time course of p65 translocation to the nucleus in PMφs with or without Chol accumulation. The mean ± SEM is plotted in all graphs. Significant differences were determined by an unpaired Student *t* test (A and B) or a two-way ANOVA and a Bonferroni post hoc test (C). *p < 0.05, **p < 0.01, ***p < 0.001, ****p < 0.0001. +, with; −, without.

Previously, we showed that oxLDL accumulation in PMφs does not inhibit LPS-induced MAPK or NF-κB signaling (5). We now show that the LPS-induced early glycolytic response also remains intact in PMφs with accumulated oxLDL (Fig. 1). Considering this, we asked whether inflammatory gene expression is reduced at the 3 h time point after LPS stimulation. Indeed, we found the mRNA expression of many inflammatory genes was reduced significantly (Fig. 5A). For comparison, we also evaluated the effects of cholesterol accumulation and found a similar phenotype (Fig. 5B). Because we showed that cholesterol accumulation in PMφs inhibits LPS-induced TBK1, AKT1, AKT2, and ACLY activation (Figs. 1–3), we assessed whether MAPK and NF-κB signaling, which are critical for the induction of many inflammatory genes, is also inhibited by cholesterol. We found that LPS-induced phosphorylation of ERK1/2 and p65 (RelA) is suppressed in PMφs with accumulated cholesterol, and nuclear translocation of p65 appears to be reduced at the 3 and 6 h time points (Fig. 5C). These data suggest that cholesterol accumulation in Mφs inhibits multiple proximal signaling pathways triggered by TLR4 stimulation, including the MAPK, NF-κB, and the PI3K/AKT pathways, and this likely contributes to suppression of inflammatory gene expression. In contrast, oxLDL accumulation in PMφs selectively inhibits AKT2 and ACLY but does not inhibit LPS-induced NF-κB or MAPK signaling or early glycolysis. Because inflammatory gene expression by PMφs with accumulated oxLDL is suppressed 3 h after LPS stimulation (Fig. 5A), this implicates AKT2 and ACLY signaling in the inhibition of inflammation at this early time point.

### oxLDL accumulation in PMφs suppresses LPS-induced acetylation of H3K27

**FIGURE 6.**
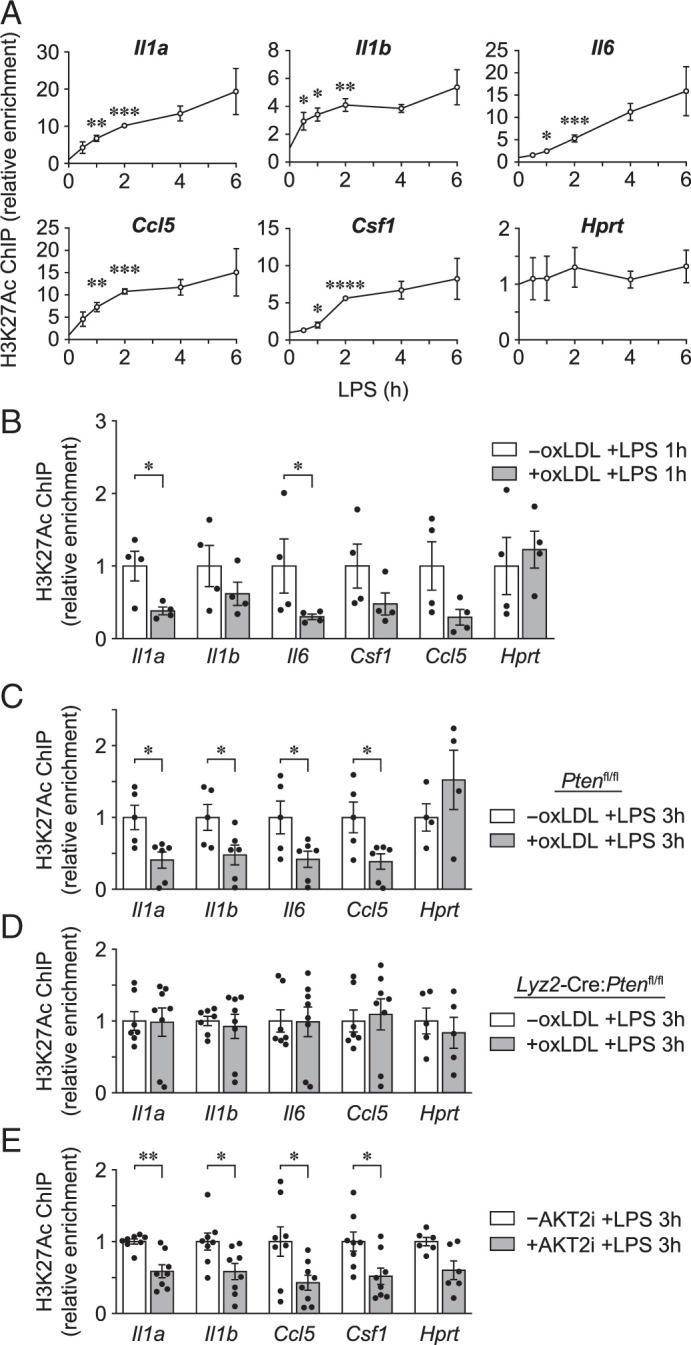
oxLDL accumulation in PMφs suppresses LPS-induced H3K27ac. ChIP-PCR experiments measuring H3K27ac enrichment in promoters of inflammatory genes. (**A**) Time course after LPS stimulation of PMφs. For each gene, the data were normalized to the 0 h LPS time point (assigned a value of 1, *n* = 3). (**B**) PMφs with or without oxLDL accumulation were assayed 1 h after LPS stimulation. For each gene, the data were normalized to the without (−)oxLDL group (assigned a value of 1, *n* = 4). (**C** and **D**) Bone marrow–derived Mφs from (C) *Pten^fl/fl^* and (D) *Lyz2-*Cre:*Pten^fl/fl^* mice with or without oxLDL accumulation were assayed 3 h after LPS stimulation. For each gene, the data were normalized to the −oxLDL group (assigned a value of 1, *n* = 4–8). (**E**) Selective inhibition of AKT2 reproduces the H3K27ac phenotype of PMφs with accumulated oxLDL. PMφs were treated with AKT2i (3 µM) 1 h prior to LPS stimulation (3 h). For each gene, the data were normalized to the −AKT2i group (assigned a value of 1, *n* = 6–8). The mean ± SEM is plotted in all graphs. Significant differences were determined by a one-way ANOVA and Bonferroni post hoc test (A), or with an unpaired Student *t* test (B–E). *p < 0.05, **p < 0.01, ***p < 0.001, ****p < 0.0001. +, with; −, without.

AKT contributes to the regulation of LPS-induced proinflammatory gene transcription through activation of ACLY and production of acetyl-CoA, which is required for protein acetylation, including K27 of histone H3 (H3K27ac) (10). Earlier, we showed that the accumulation of oxLDL in PMφs selectively suppresses activation of AKT2, ACLY, and the production of acetyl-CoA (Fig. 2). We therefore performed ChIP experiments to investigate whether the accumulation of oxLDL suppresses H3K27ac in LPS-stimulated PMφs. The initial time course showed that within 1–2 h LPS induces H3K27ac at promoters of proinflammatory genes but not *Hprt*, a constitutively expressed gene (Fig. 6A). Subsequent ChIP experiments demonstrated that oxLDL accumulation in PMφs suppresses H3K27ac within promoters of several proinflammatory genes at 1 h (Fig. 6B) after LPS stimulation.

To investigate a causal relationship between the reduction in AKT2 activation by oxLDL accumulation and the reduction in H3K27ac of the proinflammatory genes in LPS-stimulated PMϕs, we took advantage of BMDMφs from mice with myeloid cell deletion of *Pten* (*Lyz2-*Cre:*Pten*^fl/fl^), which express reduced levels of *Pten* mRNA compared with cells obtained from to *Pten* wild-type (*Pten*^fl/fl^) littermates (Supplemental Fig. 4C). The uptake of DiI-labeled oxLDL was comparable in the two genotypes (Supplemental Fig. 4D); however, oxLDL accumulation in *Lyz2-*Cre:*Pten*^fl/fl^ BMDMφs did not inhibit LPS-induced activation of AKT2 and ACLY, in contrast to *Pten*^fl/fl^ BMDMφs (Supplemental Fig. 4E). As in wild-type PMφs (Fig. 6B), oxLDL accumulation caused a reduction in H3K27ac in *Pten*^fl/fl^ BMDMφs 3 h after LPS stimulation (Fig. 6C). In contrast, oxLDL accumulation in *Lyz2-*Cre:*Pten*^fl/fl^ BMDMφs did not suppress H3K27ac within promoters of proinflammatory genes (Fig. 6D). ChIP experiments showed that treatment of PMφs with the AKT2i suppressed H3K27ac (Fig. 6E), and this phenocopied the effects of oxLDL accumulation in wild-type Mφs (Fig. 6B, 6C).

## Discussion

In atherosclerotic lesions, Mφs take up cholesterol as cholesteryl esters in modified lipoprotein particles. This is modeled in cell culture by use of oxLDL, and scavenger receptors are critical to the internalization of oxLDL. Consistent with our past studies (5, 13), we have cultured Mφs with oxLDL and in parallel with free cholesterol to overcome the controversies related to the differences in oxLDL preparations (29) and potential signaling downstream of scavenger receptors. In fact, this parallel approach enabled us to discover how oxLDL and cholesterol accumulation differentially suppressed Mφ inflammatory responses, one of which is the differential suppression of phosphorylation cascades downstream of TLR stimulation. For instance, whereas cholesterol accumulation in Mφs suppressed LPS-induced AKT1 and AKT2 signaling, oxLDL accumulation selectively impaired LPS-induced AKT2 signaling (Fig. 2C). We hypothesized that this difference is attributed to the impairment of phosphorylation cascades upstream of AKT signaling by cholesterol, but not oxLDL accumulation. Indeed, we found that the activation of TBK1, an upstream kinase of AKT (9, 20), was suppressed in PMφs with accumulated cholesterol, but not oxLDL (Fig. 3A). The notion that cholesterol, but not oxLDL, accumulation could interfere with early phosphorylation cascades was further reinforced by experiments showing that inhibition of PTEN prior to LPS stimulation restored AKT activation in PMφs with accumulated cholesterol (Fig. 3C), but not AKT2 activation in PMφs with accumulated oxLDL (Fig. 3B). Indeed, this differential response was also found in other major LPS-induced inflammatory phosphorylation events. As shown in Fig. 5C, although cholesterol accumulation in PMφs impaired LPS-induced NF-κB and ERK activation, they remained unaffected in PMφs with accumulated oxLDL, as was shown in our past study (5). Taken together, our data demonstrate that cholesterol and oxLDL accumulation have differential effects on suppressing signaling cascades. We speculate that the inhibitory effect of cholesterol accumulation is due to a significant disruption of the cholesterol composition within the plasma membrane and lipid rafts (30). In contrast, the selective inhibitory effect of oxLDL accumulation is caused by generation of mitochondrial ROS, which modifies and inhibits AKT2.

Because phosphorylation cascades regulate downstream biological functions, it is possible that Mφs with accumulated cholesterol versus oxLDL would exhibit different biological responses to inflammatory stimuli. Although both cholesterol and oxLDL accumulation impaired LPS-induced activation of AKT (Fig. 1A, 1B), a critical kinase with pleiotropic functions including early glycolytic influx, the induction of early-phase glycolysis remains intact in PMφs with accumulated oxLDL (Fig. 1C). We attribute this to the selective impairment of AKT2 signaling and intact AKT1 activation. This result demonstrates that AKT2 activation is dispensable for LPS-induced early-phase glycolysis and was confirmed by use of a selective AKT2 inhibitor (Fig. 2E). Although AKT2 activation is not essential for glycolysis, we found that it is required for the activation of ACLY and consequently the production of acetyl-CoA (Fig. 2A, 2B, 2F, 2G). Recent research has demonstrated the importance of ACLY-derived acetyl-CoA in the regulation of Mφ inflammatory gene expression through epigenetics (10). Specifically, ACLY-derived acetyl-CoA is critical for regulating H3K27ac of LPS-induced proinflammatory gene promoters (10). In our study, we have refined this concept by providing evidence that AKT2 regulates ACLY activity, acetyl-CoA levels (Fig. 2F, 2G), and H3K27ac of proinflammatory genes (Fig. 6E). More importantly, in Mφs with accumulated oxLDL where AKT2 activation is selectively inhibited, ACLY activity and acetyl-CoA levels (Fig. 2A, 2B) and H3K27ac of proinflammatory genes were impaired (Fig. 6B, 6C). Collectively, our data suggest that oxLDL-mediated inhibition of early inflammatory responses is regulated by AKT2 and ACLY, instead of glycolytic enzymes. An important question that remains to be answered is whether oxLDL-induced reduction of H3K27ac is directly linked to the suppression of proinflammatory gene expression (Fig. 5A). Our previous study has shown that the binding of the prototypic p65 (RelA) subunit of NF-κB to the promoters of key proinflammatory genes, such as *Ccl5* and *Il6*, was impaired in Mφs with accumulated oxLDL, and that blocking histone deacetylase activity partially restored the expression of these genes (5). Because histone acetylation regulates the accessibility of transcription factors binding to promoters of target genes, this raises the possibility that suppression of H3K27ac by oxLDL accumulation (Fig. 6B) impairs the binding of p65 within 3 h of LPS stimulation.

Apart from p65, other lipid sensing transcription factors, such as liver X receptors (LXRs), peroxisome proliferator-activated receptors (PPARs), and retinoid X receptors (RXRs) have been implicated in mediating the suppressive inflammatory responses found in lipid loaded Mφs. For instance, cholesterol loading-induced desmosterol accumulation was shown to activate LXRs and suppress inflammation in Mφ foam cells (31). These findings were subsequently confirmed in foam cells derived from atherosclerotic lesions, as deletion of LXRs in myeloid cells accelerated atherosclerosis (32). Similar to LXRs, in vitro studies have shown that agonists of PPARs could block LPS-induced Mφ activation and secretion of inflammatory cytokines due to inhibition of inflammatory transcription factor functions (33). The possible antiatherogenic role of PPARs was then confirmed with in vivo atherosclerotic mouse models (34). Finally, agonists for RXRs have also been shown to be critical for mediating the suppressive inflammatory responses in Mφs, especially in chronic inflammatory disease models, such as atherosclerosis (35). Because RXRs have been recently shown to regulate the maintenance and identity of large PMφs (36), it also raises the possibility that RXRs could mediate the reduced inflammatory responses that we have observed in oxLDL-loaded PMφs.

We noted that oxLDL accumulation in PTEN-deficient Mϕs did not suppress LPS-induced AKT2 and ACLY activation (Supplemental Fig. 4E), unlike the effects of PTENi that was added 1 h before LPS treatment and after oxLDL-induced ROS production (Figs. 3B, 4D). These observations support the notion that oxLDL/ROS-mediated inhibition of AKT2 occurs prior to the addition of PTENi. In contrast, PTEN-deficient *Lyz2-*Cre:*PTEN*^fl/fl^ Mϕs have constitutively increased PIP_3_ and AKT activation and appear to be resistant to oxidative stress induced by oxLDL accumulation.

In addition to the reduction of ACLY activation, we have also observed that oxLDL loading of LPS-activated Mϕs led to the suppression of mTORC1 activation downstream of AKT2 signaling (Supplemental Fig. 1C). Past research has shown in adipocytes that AKT2, specifically its phosphorylation on Ser^474^, is critical for insulin-mediated mTORC1 activation (37). This raises the possibility that oxLDL-mediated inhibition of AKT2 led to the suppression of mTORC1 signaling cascades, including protein synthesis. Because the mRNA of HIF-1α is particularly sensitive to mTORC1 activation, as it harbors a 5′ terminal oligopyrimidine tract (38), oxLDL-mediated inhibition of AKT2 activity may thus reduce the translation of HIF-1α mRNA, and potentially contribute to the impaired HIF-1α expression observed in Mφs with accumulated oxLDL during the late phase of glycolytic reprogramming (13).

oxLDL-induced ROS production plays a critical role in shaping how foam cells respond to inflammatory stimuli. For instance, although others have shown that oxLDL-induced ROS is a rapid NADPH oxidase–dependent event resulting from inflammatory signaling (39), we found in this study, as well as previously (13), that oxLDL-induced ROS production is a relatively late event. This suggests that the production of ROS induced by oxLDL accumulation is a consequence of a metabolic adaptation in the mitochondria. Indeed, our transcriptomic data and qPCR analysis demonstrate an upregulation of fatty acid oxidation in PMφs with accumulated oxLDL (Fig. 4A, 4C). This is significant not only because it demonstrates that cells with lipid accumulation acquire a new metabolic adaptation, but more importantly, ROS generated as part of this adaptation may modulate the response upon subsequent stimulation with inflammatory agonists. For instance, we have previously shown that oxLDL-induced ROS primed an enhanced LPS-induced NRF2-dependent antioxidative response that suppressed HIF-1α–dependent late-phase glycolysis and inflammation (13). Now we show that oxLDL-induced mitochondrial ROS suppress LPS-induced activation of AKT2 and ACLY and thus reduce H3K27ac of inflammatory genes. Taken together, our previous and current studies collectively demonstrated that the generation of ROS in Mφs with accumulated oxLDL plays an important role in modulating epigenetic and transcriptional responses to inflammatory stimuli.

AKT2 genetic deficient models from in vitro and in vivo studies have established its role in mediating cellular metabolism and inflammation (15, 22), yet how AKT2 can be regulated independently from other isoforms is not well understood. In recent years, the importance of cysteine 124 (C124) of AKT2 in regulating its catalytic function has been demonstrated (26, 27). C124 of AKT2 is a highly conserved residue only found in AKT2, but not other isoforms (26). Due to its position within the linker region, and its highly reactive nature to oxidative radicals, it is postulated that ROS are involved in AKT2-specfic regulation. Indeed, several studies have now demonstrated that the oxidation of C124 is critical in regulating AKT2 activity in response to oxidative stress, thereby linking redox homeostasis with the regulation of phosphorylation cascades (26, 27). In our study, inhibition of mitochondria-derived ROS during oxLDL accumulation reinstated AKT2 activation (Fig. 4F), thereby supporting the above notion that changes in the redox environment can induce AKT2-specifc regulations, and that the oxidation of C124 by oxLDL-induced ROS may explain how oxLDL can selectively inactivate AKT2 but not other isoforms.

In this study we used thioglycolate-elicited monocyte-derived PMφs to model arterial intimal Mφs in atherosclerotic lesions, which are derived primarily from blood monocytes. Key findings were reproduced in BMDMφs and RAW264.7 cells. However, it is possible that in other Mφs, such as tissue-resident Mφs, intracellular lipid accumulation could elicit different responses upon exposure to inflammatory stimuli.

Taken together, our study shows that the accumulation of oxLDL or cholesterol in Mφs impairs LPS-induced AKT signaling. Unlike cholesterol, oxLDL loading selectively impairs the activation and activity of AKT2, but not other isoforms, due to mitochondria-derived ROS. This impairment subsequently led to the reduction of ACLY activity, acetyl-CoA levels, and H3K27ac of proinflammatory genes, independent of LPS-induced early-phase glycolysis.

## Supplementary Material

Supplemental Figures 1 (PDF)
