# Supplementary material for: Oxidized Low-Density Lipoprotein Accumulation in Macrophages Impairs Lipopolysaccharide-Induced Activation of AKT2, ATP Citrate Lyase, Acetyl–Coenzyme A Production, and Inflammatory Gene H3K27 Acetylation
Source: Immunohorizons. 2024 Jan 9;8(1):57–73. doi: 10.4049/immunohorizons.2300101 (PMC10835650; doi:10.4049/immunohorizons.2300101)
Supplement: Supplemental Figures 1 (PDF) [file IH_2300101_Supplemental_1.pdf]

Supplemental Figure 1

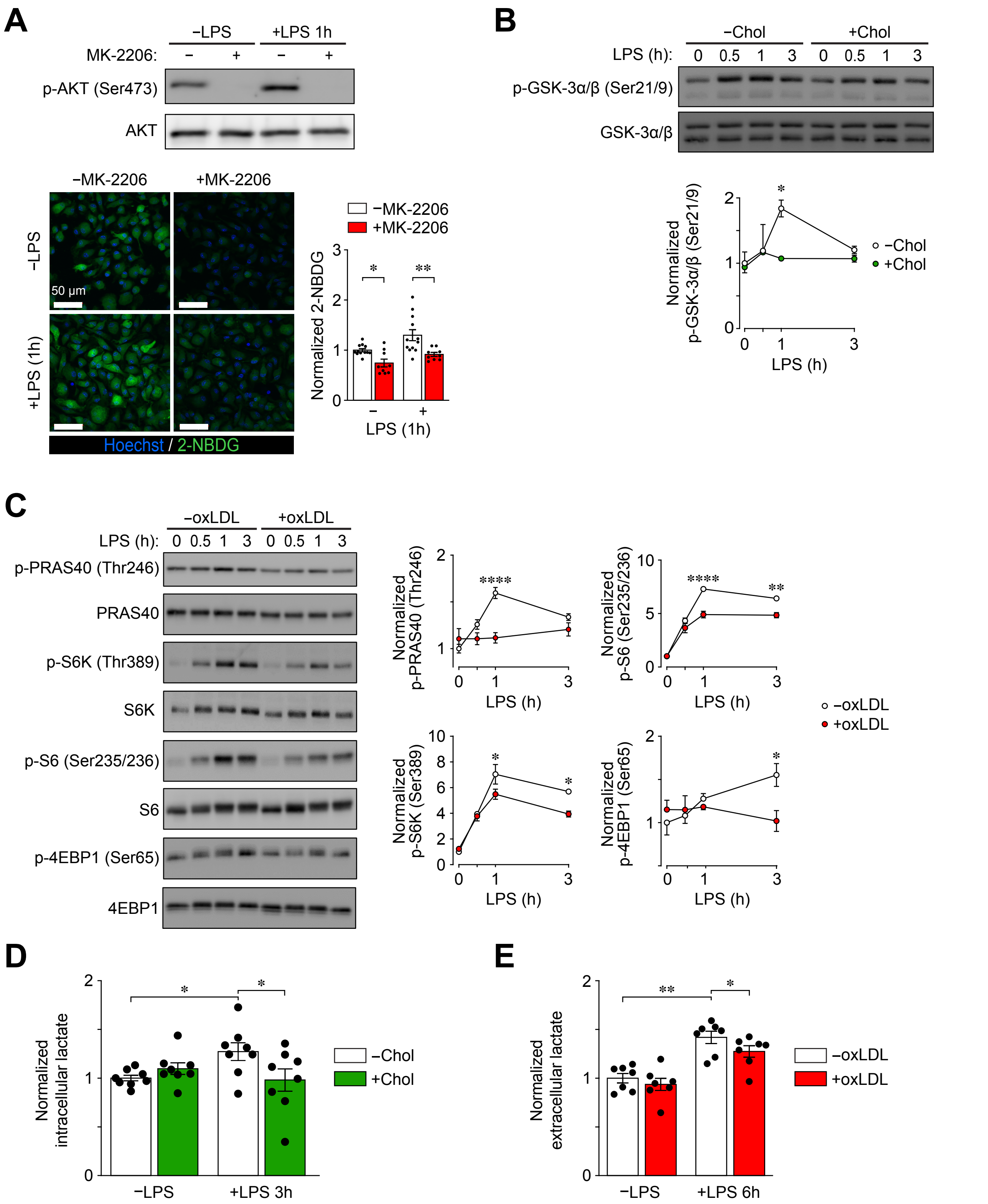

(A) Representative immunoblots (top) of p-AKT (Ser473) and AKT in PMφs with or without MK-2206 treatment and LPS stimulation (1 hour). Representative confocal microscope images and quantification (bottom) of 2-NBDG uptake (green) in PMφs with or without MK-2206 treatment and LPS stimulation (1 hour) (n = 9-12). **(B)** Representative immunoblots and quantification of p-GSK-3α/β (Ser21/9) and GSK-3α/β in PMφs with and without Chol accumulation and LPS stimulation (n = 2). **(C)** Representative immunoblots and quantification of p-PRAS40 (Thr246), PRAS40, p-S6K (Thr389), S6K, p-S6 (Ser235/236), S6, p-4EBP1 (Ser65) and 4EBP1 in PMφs with and without oxLDL accumulation and LPS stimulation (n = 3-5). **(D)** Normalized intracellular lactate in PMφs with and without Chol accumulation and 3 hours of LPS stimulation (n = 8). **(E)** Normalized extracellular lactate in cultures of PMφs with and without oxLDL accumulation and 6 hours of LPS stimulation (n = 7). The mean ± SEM is plotted in all graphs. Statistically significant differences were determined by a two-way ANOVA and a Bonferroni post hoc test. *$p < 0.05$, **$p < 0.01$, ****$p < 0.0001$. +, with; −, without.

Supplemental Figure 2

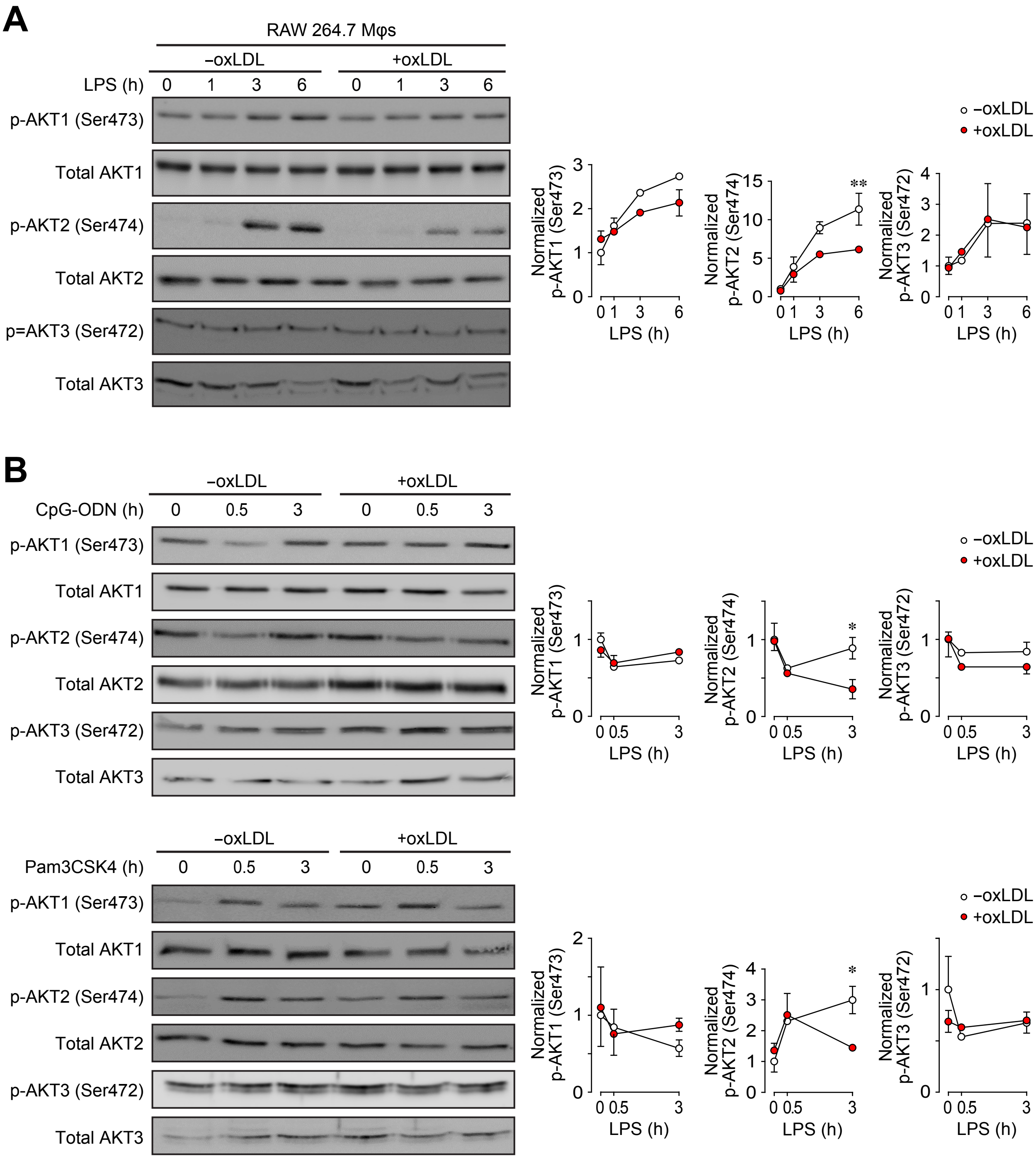

(A) Representative immunoblots and quantification of p-AKT1 (Ser473), AKT1, p-AKT2 (Ser474), AKT2 and p-AKT3 (Ser472) and AKT3 in RAW264.7 Mφs with and without oxLDL accumulation and LPS stimulation (n = 3). **(B)** Representative immunoblots and quantification of p-AKT1 (Ser473), AKT1, p-AKT2 (Ser474), AKT2, p-AKT3 (Ser472), AKT3 in PMφs with and without oxLDL accumulation and CpG or Pam3CSK4 stimulation (n = 3). The mean ± SEM is plotted in all graphs. Statistically significant differences were determined by a two-way ANOVA and a Bonferroni post hoc test. **p* < 0.05, ***p* < 0.01. +, with; −, without.

Supplemental Figure 3

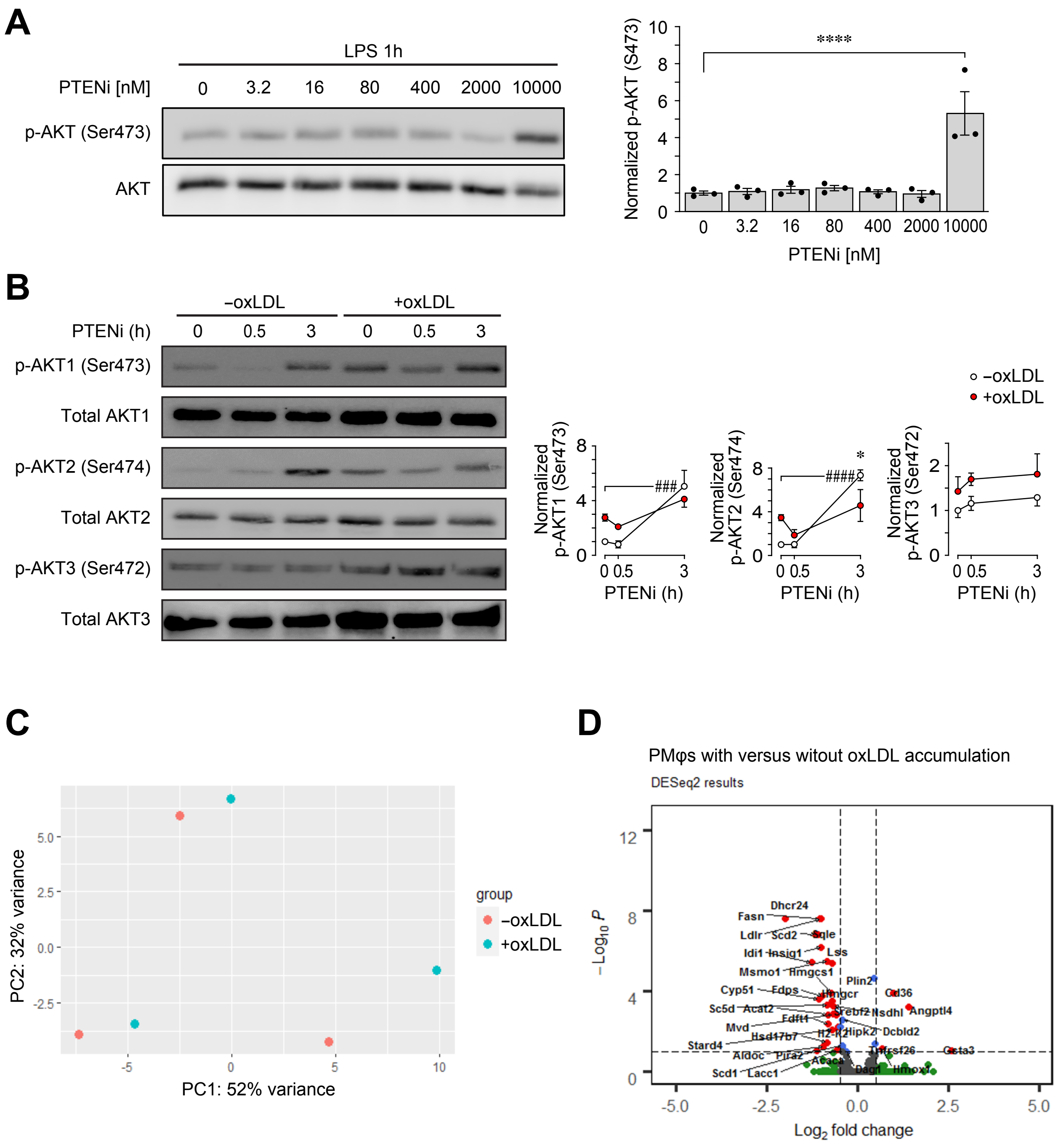

(A) Representative immunoblots and quantification of p-AKT (Ser473) and AKT in PMφs treated with increasing concentrations of PTENi added 1 hour prior to stimulation with LPS for 1 hour (n = 3). **(B)** Representative immunoblots and quantification of p-AKT1 (Ser473), AKT1, p-AKT2 (Ser474), AKT2, p-AKT3 (Ser472), AKT3 in PMφs with and without oxLDL accumulation and treatment with PTENi for up to 3 hours (n = 3). The asterisk indicates a significant difference between PMφs with and without oxLDL accumulation at the same time point (3 hours) after treatment with PTENi. Number symbols indicate significant difference between 0 and 3 hours of PTENi treatment of PMφs without oxLDL accumulation. **(C, D)** Principal component analysis of sample variance (C, n = 3) and volcano plot (D) illustrating enrichment of genes in PMφs with oxLDL versus without oxLDL accumulation. The mean ± SEM is plotted in all graphs. Statistical significance was determined by a one-way ANOVA (A) or a two-way ANOVA (B) and a Bonferroni post hoc test. *$p < 0.05$, ****$p < 0.0001$, ###$p < 0.001$, ####$p < 0.0001$. +, with; −, without.

Supplemental Figure 4

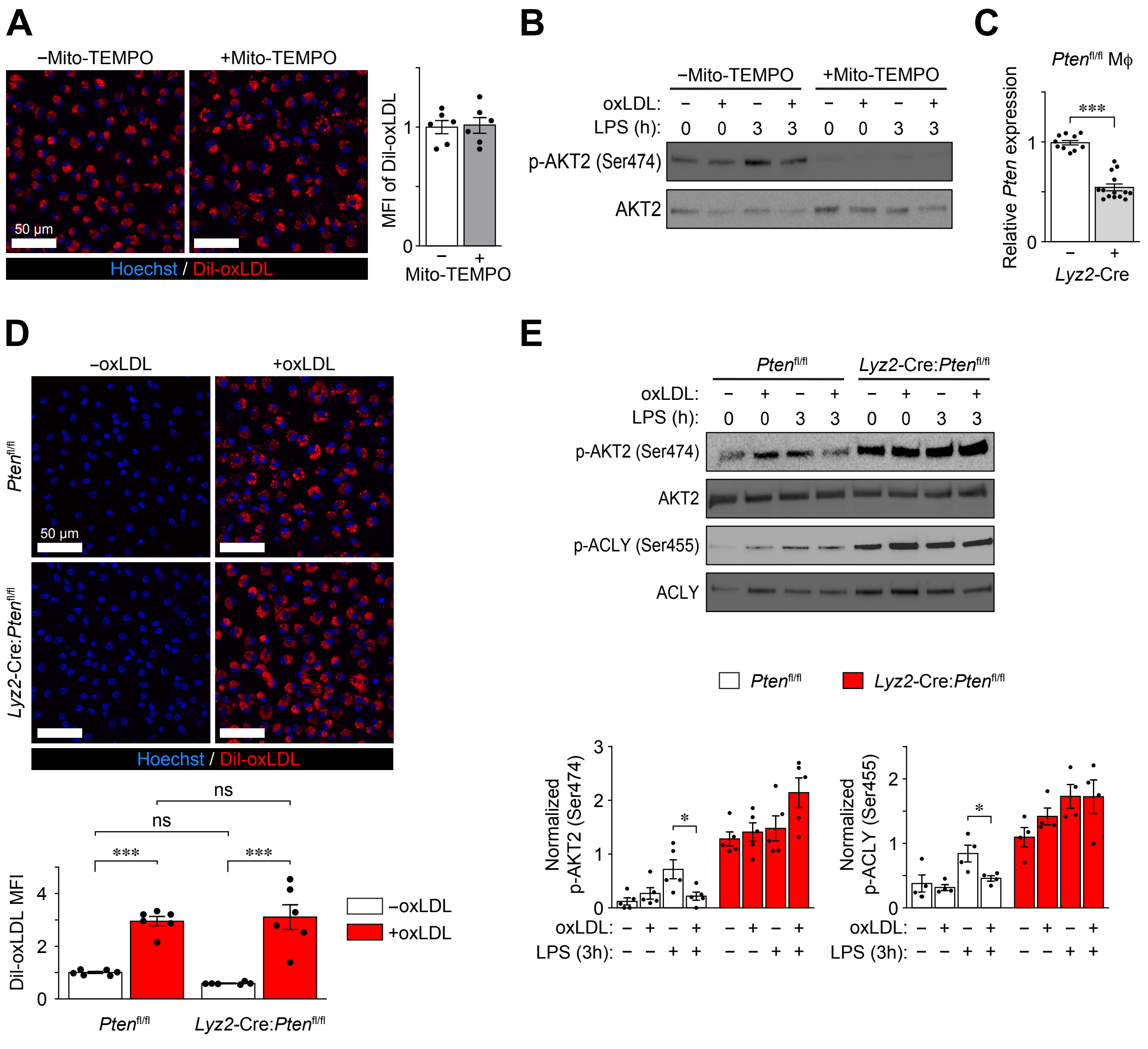

(A) Representative confocal microscope images and quantification of DiI-oxLDL accumulation in PMφs cultured with oxLDL and pretreated with or without Mito-TEMPO (n = 6). **(B)** Immunoblot analysis of AKT2 activation (phosphorylation of serine-474) in PMφs with and without Mito-TEMPO treatment, oxLDL accumulation and 3 hours of LPS stimulation (n = 1). The blot shows that pretreatment with Mito-TEMPO inhibits AKT2 phosphorylation. **(C)** qPCR analysis of *Pten* mRNA expression in bone marrow derived Mφs from *Pten*$^{fl/fl}$ and *Lyz2*-Cre:*Pten*$^{fl/fl}$ mice (n = 11-14). **(D)** Representative confocal microscope images and quantification of DiI-oxLDL accumulation in bone marrow derived Mφs from *Pten*$^{fl/fl}$ and *Lyz2*-Cre:*Pten*$^{fl/fl}$ mice cultured with and without oxLDL (n = 6). **(E)** Representative immunoblots and quantification of p-AKT2 (Ser474), AKT2, p-ACLY (Ser455) and ACLY in bone marrow derived Mφs from *Pten*$^{fl/fl}$ and *Lyz2*-Cre:*Pten*$^{fl/fl}$ mice with and without oxLDL accumulation and 3 hours of LPS stimulation (n = 4-5). The mean ± SEM is plotted in all graphs. Significant differences were determined by an unpaired Student's t-test (A, C) or a two-way ANOVA and a Bonferroni post hoc test (D, E). *$p < 0.05$, ***$p < 0.001$, ns, not significant. +, with; –, without.
